# Running a Marathon Induces Changes in Adipokine Levels and in Markers of Cartilage Degradation – Novel Role for Resistin

**DOI:** 10.1371/journal.pone.0110481

**Published:** 2014-10-21

**Authors:** Katriina Vuolteenaho, Tiina Leppänen, Riina Kekkonen, Riitta Korpela, Eeva Moilanen

**Affiliations:** 1 The Immunopharmacology Research Group, University of Tampere School of Medicine and Tampere University Hospital, Tampere, Finland; 2 Institute of Biomedicine, Pharmacology, University of Helsinki, Helsinki, Finland; SERGAS, Santiago University Clinical Hospital, IDIS Research Laboratory 9, NEIRID Lab, Spain

## Abstract

Running a marathon causes strenuous joint loading and increased energy expenditure. Adipokines regulate energy metabolism, but recent studies have indicated that they also exert a role in cartilage degradation in arthritis. Our aim was to investigate the effects of running a marathon on the levels of adipokines and indices of cartilage metabolism. Blood samples were obtained from 46 male marathoners before and after a marathon run. We measured levels of matrix metalloproteinase-3 (MMP-3), cartilage oligomeric protein (COMP) and chitinase 3-like protein 1 (YKL-40) as biomarkers of cartilage turnover and/or damage and plasma concentrations of adipokines adiponectin, leptin and resistin. Mean marathon time was 3∶30∶46±0∶02∶46 (h:min:sec). The exertion more than doubled MMP-3 levels and this change correlated negatively with the marathon time (r = –0.448, p = 0.002). YKL-40 levels increased by 56% and the effect on COMP release was variable. Running a marathon increased the levels of resistin and adiponectin, while leptin levels remained unchanged. The marathon-induced changes in resistin levels were positively associated with the changes in MMP-3 (r = 0.382, p = 0.009) and YKL-40 (r = 0.588, p<0.001) and the pre-marathon resistin levels correlated positively with the marathon induced change in YKL-40 (r = 0.386, p = 0.008). The present results show the impact of running a marathon, and possible load frequency, on cartilage metabolism: the faster the marathon was run, the greater was the increase in MMP-3 levels. Further, the results introduce pro-inflammatory adipocytokine resistin as a novel factor, which enhances during marathon race and associates with markers of cartilage degradation.

## Introduction

Moderate recreational activity is an effective mean to improve health showing many long-term benefits: improved lung and heart function, muscle strength and metabolic health among other things. However, after intensive exercise, such as marathon, markers of muscle damage, oxidative stress and inflammation are often found [1], [2]. Running has beneficial effects on knee articular cartilage, but elite athletes who perform their activities with high impacts and exert high stresses on their joints appear to have increased risk to develop osteoarthritis and the concomitant presence of joint injury further increases this risk [3]. The effects of joint loading caused by exercise were investigated in the recent MRI study by Mosher et al. [4]. Those authors found that there was measurable compression of cartilage after 30 min of running. There are also reports on the effects of joint loading and exercise on the release of biomarkers of cartilage turnover and/or damage focusing mainly on COMP but little is known about more recent biomarkers MMP-3 and YKL-40 which are known to associate with cartilage degradation in inflammatory arthritis and/or osteoarthritis [5]–[13].

Adipokines (also known as adipocytokines) adiponectin, leptin and resistin, are hormones that were initially found to be secreted by adipocytes and to regulate energy metabolism linking nutritional status to neuroendocrine function [14]. Interestingly, more recent findings on the ubiquitous expression of their receptors and on their cellular effects have revealed that adipokines are produced by joint tissues and can act as regulatory factors in inflammatory arthritis as well as in osteoarthritis. We and others have recently shown that those adipokines are especially associated with cartilage destruction in osteoarthritic joints [15]–[18]. Running a marathon or some other long-duration exercises have been reported either to decrease or not to have any effect on leptin levels [19]–[22], but as far as we are aware the effects of marathon running on adiponectin and resistin levels have not previously been investigated. Further, the association of adipokines with cartilage degradation in relation to strenuous exercise like marathon run has not been previously studied although such a relationship could be hypothesized based on osteoarthritis studies.

The aim of the present study was to investigate the effects of marathon running on the levels of adipokines adiponectin, leptin and resistin, as well as on markers associated with cartilage degradation in inflammatory arthritis and osteoarthritis; i.e. COMP, MMP-3 and YKL-40, in an attempt to further understand the effects of running associated strenuous loading on articular cartilage. The present results highlight the impact of long distance running on cartilage metabolism and imply that load frequency increases cartilage turnover/damage: the faster the marathon was run, the greater the increase in the MMP-3 level. Also YKL-40 levels were increased, while the effect on COMP was variable. Further, the results introduce the proinflammatory adipocytokine resistin as a novel factor increased during strenuous exercise and associated with markers of cartilage degradation.

## Methods

### Subjects

The subjects were recruited among those planning to participate in the Helsinki City Marathon (42.195 km) through an advertisement in a national runners’ magazine and by sending a recruitment letter to previous Helsinki City Marathon participants. The subjects were eligible for the study if they were healthy and their personal-best marathon time was less than 3 h 30 min. Because of gender-related differences in adipokine concentrations, only males were included in the study. The subjects provided their written informed consent before entering the study. During the marathon, the subjects were permitted to ingest fluids and food freely. The study was approved by the Ethics Committee of the Hospital District of Helsinki and Uusimaa, Finland and was conducted according to the principles expressed in the Declaration of Helsinki.

### Blood samples

Venous blood samples were taken from the antecubital vein into EDTA tubes on the day before the marathon and after the race. Blood samples were taken in the Helsinki Olympic Stadium before and after the marathon run, sent immediately to the laboratory, centrifuged and the resulting plasma stored as aliquots at −80°C until analyzed.

### Laboratory analyses

Concentrations of adiponectin, leptin, resistin, MMP-3, COMP and YKL-40 in plasma samples were determined by enzyme-linked immunosorbent assay (ELISA) with commercial reagents (adiponectin, leptin, resistin, MMP-3 and YKL-40: R&D Systems, Europe Ltd, Abindgon, UK; COMP: Biovendor, Modrice, Czech Republic). Hematological parameters were determined using an electronic counter (Coulter MAXM hematology analyzer, Beckman Coulter, Fullerton, CA). Changes in plasma volume due to the marathon run were estimated using hematological parameters as described by Dill and Costill [23] and results presented were corrected for changes in plasma volume.

### Statistical analysis

Data were analyzed using SPSS 19.0 software for Windows (SPSS Inc., Chicago, IL, USA). The results are presented as median and mean, interquartile range, minimum and maximum score, and outliers, unless otherwise indicated. The normality of the distributions of the analytes were tested with Kolmogorov-Smirnov’s test. Changes in plasma levels of adipokines and biomarkers before and after the marathon were analysed with two-tailed paired t-test or Wilcoxońs test, where appropriate. Pearson and Spearman correlation analysis were used when appropriate according to the distribution of the data. P-values less than 0.05 were considered as statistically significant.

## Results

Forty six (46) men (Table 1) completed the marathon and were included in the study. marathon time was 3∶30∶46±0∶02∶46 h:min:sec (mean±SEM). The mean change in plasma volume due to the marathon run was −1.7±0.9% and thus the results presented below are corrected for changes in plasma volume.

**Table 1:** Demographic data on the 46 male marathoners participating in the study.

Characteristic	Mean	SD
**Age, years**	40.3	9.9
**Body-mass index, kg/m^2^**	22.3	1.2
**Marathon time h:min:sec**	3∶30∶46	0∶18∶47
**Hemoglobin, g/l**	147.6	7.8
**Hematocrit, %**	44.0	2.5

**Figure 1:**
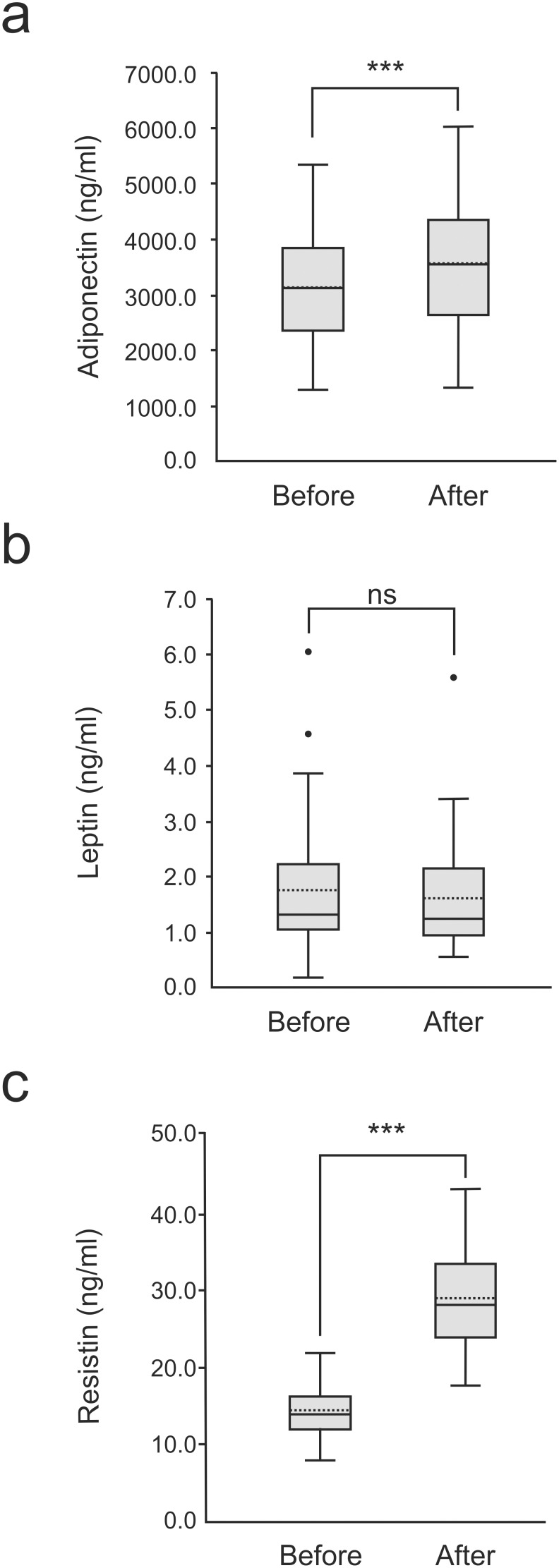
Adipokine levels before and after the marathon race. Horizontal solid and dashed bars within the boxes represent median and mean, respectively, boxes represent interquartile range and lines outside boxes represent minimum and maximum. Outliers are indicated. Paired t-test or Wilcoxońs test was used where appropriate. ***  =  p<0.001, n = 46.

The adiponectin level measured before the marathon race was 3142±145 ng/ml; leptin concentration 1.7±0.2 ng/ml, and resistin concentration 13.7±0.5 ng/ml. None of the adipokines showed correlation to BMI at baseline. After the marathon race, resistin levels had been increased by 107% (p<0.001) and those of adiponectin by 13% (p<0.001), but there was no change in the leptin levels (Fig. 1).

**Figure 2:**
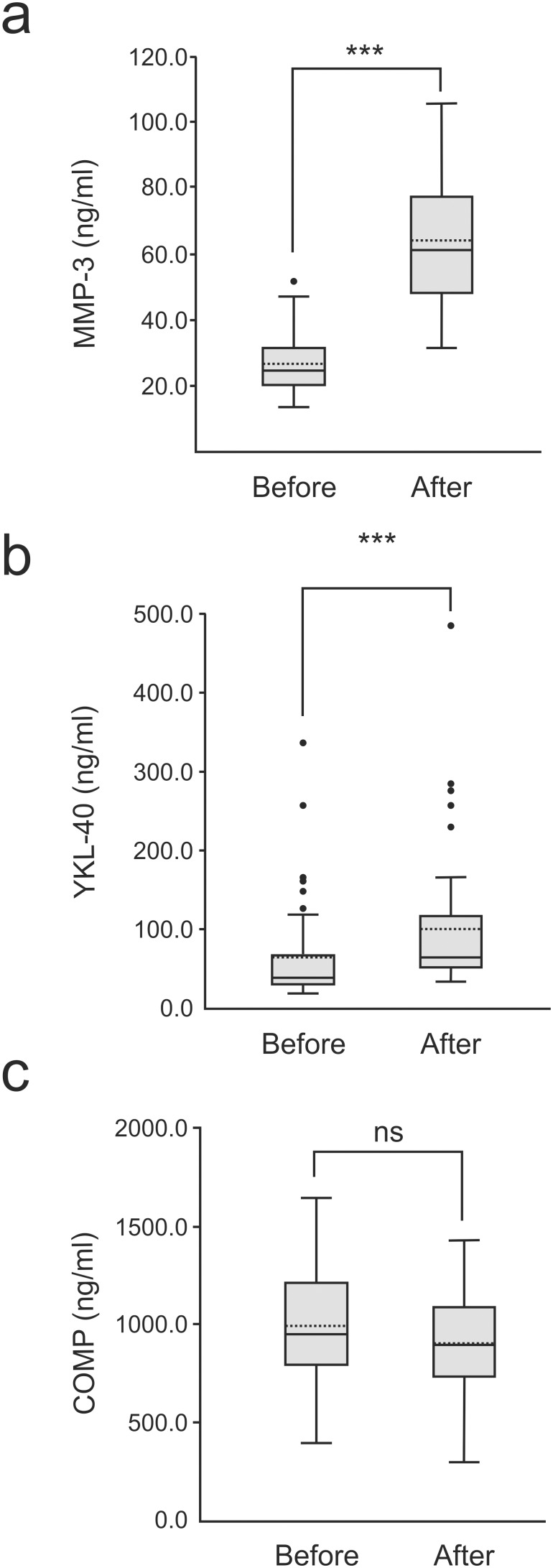
MMP-3, YKL-40 and COMP as a measure of cartilage metabolism before and after the marathon. Horizontal solid and dashed bars within boxes represent median and mean, respectively, boxes represent interquartile range and lines outside boxes represent minimum and maximum. Outliers are indicated. Paired t-test or Wilcoxońs test was used where appropriate. ***  =  p<0.001, n = 46.

The pre-marathon level of MMP-3 was 26.6±1.1 ng/ml and running the marathon more than doubled the MMP-3 concentration (142% increase, p<0.001, Fig. 2a). The YKL-40 level was 63.8±9.5 ng/ml before the marathon race and after the run YKL-40 level had increased by 56% (p<0.001, Fig. 2b). Before the marathon, the COMP concentration was 996.2±44.4 ng/ml and the mean level of COMP showed no change after the race (Fig. 2c).

**Figure 3:**
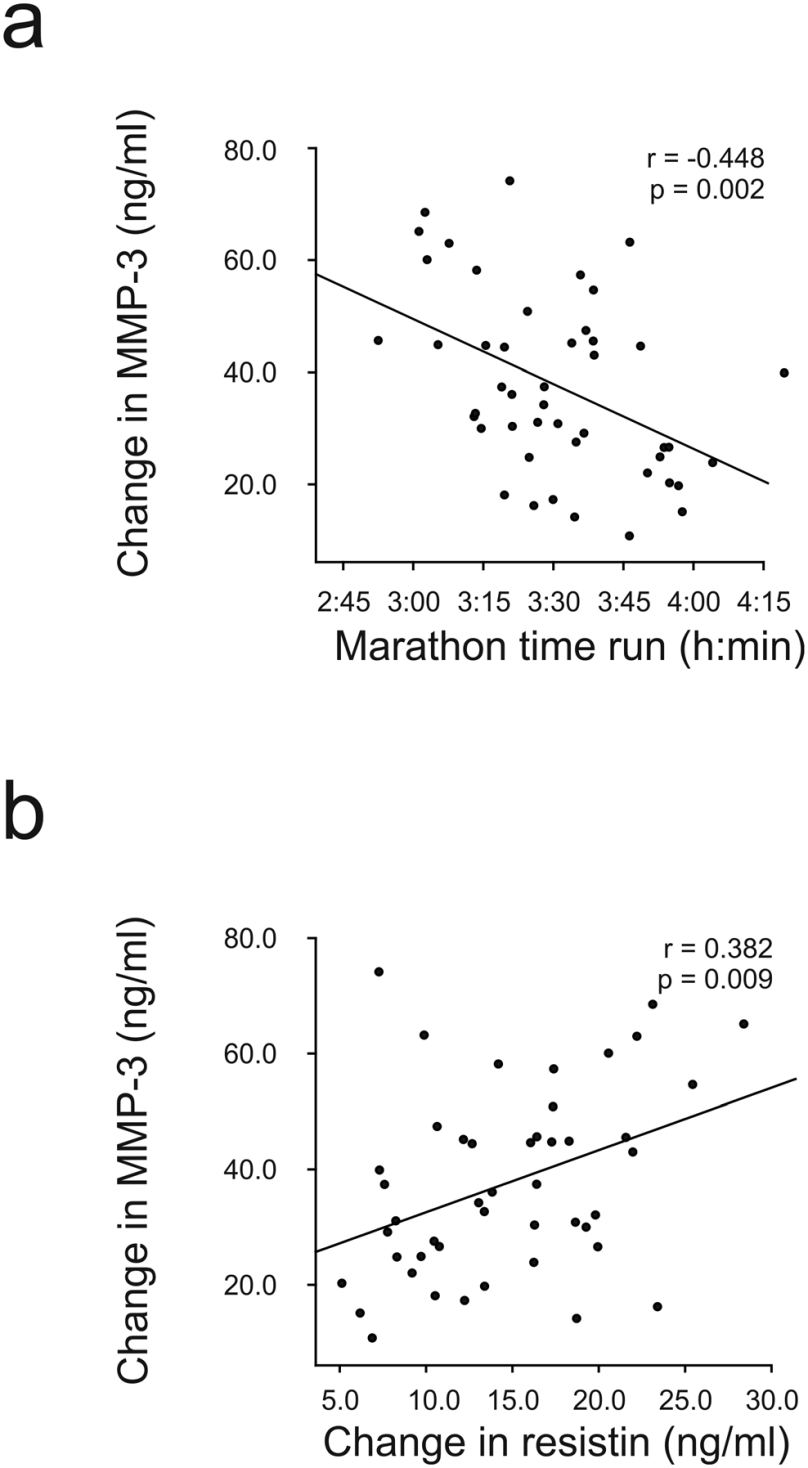
Associations of change in MMP-3 level with marathon time and change in resistin concentration. Marathon induced change in MMP-3 level was negatively correlated with marathon time (a), i.e. the faster the marathon was run the more MMP-3 level increased due to the marathon. Change in MMP-3 level correlated positively with change in resistin concentration (b). Pearson or Spearman correlation analyses were used where appropriate, n = 46.

MMP-3 levels were increased in all of the marathoners and furthermore, the marathon induced change in the MMP-3 concentration was negatively correlated with the marathon time (r  =  −0.448, p = 0.002, Fig. 3a), i.e. the faster the marathon was run, the greater the increase in the MMP-3 level. Interestingly, the increase detected in resistin levels was associated positively with the increase in MMP-3 levels (r = 0.382, p = 0.009, Fig. 3b).

**Figure 4:**
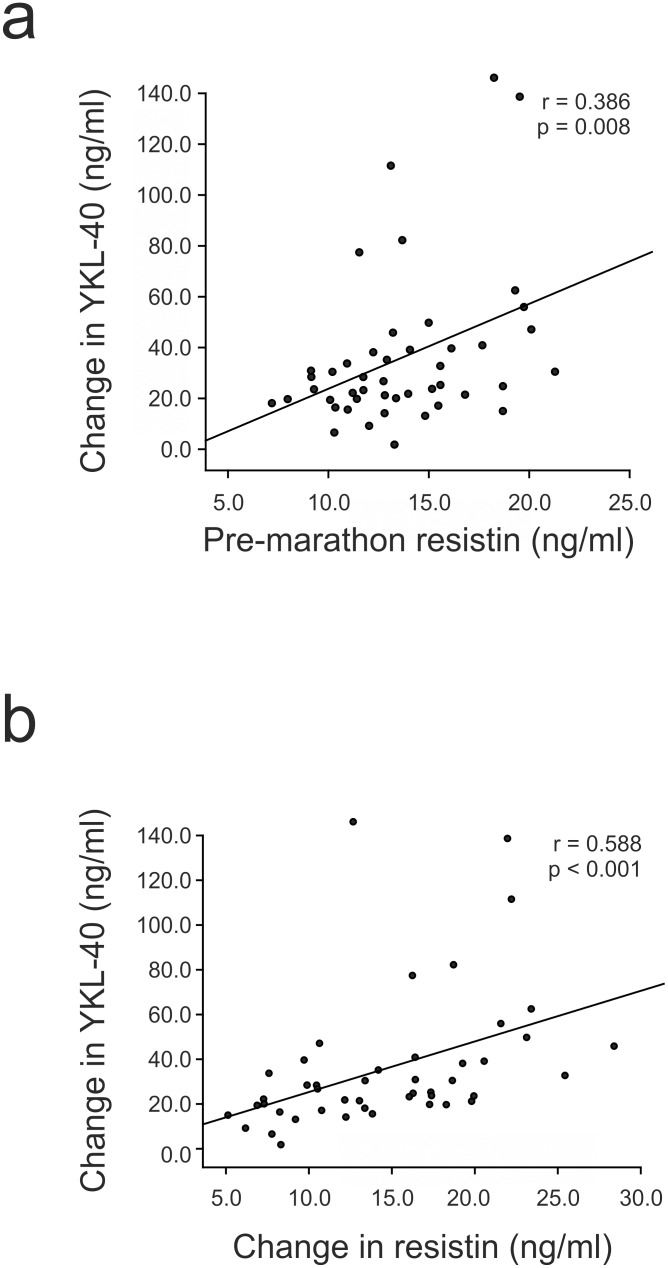
Associations of change in YKL-40 with pre-marathon level of resistin and change in resistin. Marathon induced change in YKL-40 was positively correlated with pre-marathon level of resistin, i.e. high baseline resistin predicted large increase in YKL-40 levels after the marathon (a). Change in YKL-40 level was also positively correlated with change in resistin concentration (b). Pearson or Spearman correlation analyses were used where appropriate, n = 46.

The marathon increased YKL-40 levels in all of the runners. Pre-marathon resistin levels predicted the change in YKL-40 levels (r = 0.386, p = 0.008, Fig. 4a) and, interestingly, the marathon induced increase in resistin levels correlated positively with the elevation of the YKL-40 concentrations (r = 0.588, p<0.001, Fig. 4b).

Although the average COMP levels did not change (Fig. 2), there was variation between the runners; maximum individual increase in COMP was 493.6 ng/ml, while it was decreased in some subjects. Low adiponectin and high leptin pre-marathon levels predicted a greater increase in COMP concentration (r  =  −0.365, p = 0.013 for adiponectin and r = 0.528, p<0.001 for leptin, Table 2).

**Table 2:** Associations of adipokines and marathon time run with marathon induced changes in MMP-3, YKL-40 and COMP as indices of cartilage metabolism.

			Marathon induced change in		
			MMP-3	YKL-40	COMP
**Marathon time run**		r =	**–0.448**	–0.191	0.029
		p =	**0.002**	0.205	0.848
**Adiponectin**	pre-marathon level	r =	–0.067	–0.082	**–0.365**
		p =	0.657	0.586	**0.013**
	marathon induced change	r =	0.129	0.047	0.038
		p =	0.392	0.759	0.802
**Leptin**	pre-marathon level	r =	0.130	0.118	**0.528**
		p =	0.390	0.433	**<0.001**
	marathon induced change	r =	0.071	–0.065	–0.192
		p =	0.640	0.666	0.201
**Resistin**	pre-marathon level	r =	0.007	**0.386**	–0.113
		p =	0.965	**0.008**	0.454
	marathon induced change	r =	**0.382**	**0.588**	0.257
		p =	**0.009**	**<0.001**	0.085

Pearson and Spearman correlation analysis were used when appropriate according to the distribution of the data. n = 46.

## Discussion

Although moderate exercise is beneficial for the joint and articular cartilage [3], marathon running is suspected to exert unusually strenuous joint loading. We detected clear increases in known biomarkers of cartilage turnover and/or damage: MMP-3 levels increased by 142% and YKL-40 levels by 56%. In addition, the marathon induced change in the MMP-3 level was negatively correlated with the marathon time (r  =  −0.448, p = 0.002), i.e. the faster the marathon was run, the greater the increase in the MMP-3 level.

In arthritis research, increased levels of MMP-3, YKL-40 and COMP in the circulation are recognized as biomarkers of cartilage turnover and/or destruction [24]. MMP-3 is a protease stromelysin produced by synovial tissue and cartilage and involved in cartilage matrix degradation and inflammation inside joints. YKL-40 is a glycoprotein belonging to the chitinase family secreted by cartilage, synovium, macrophages and cells of epithelial origin and involved in cartilage catabolism [24]. COMP is a cartilage matrix component, which is known to be released during cartilage degeneration [10], [24]. To our knowledge, there are no previous reports on the effect of running a marathon on MMP-3 or YKL-40 levels. In the present study, the strenuous exercise of a marathon race more than doubled the circulating levels of MMP-3 and this correlated negatively with the time to complete the race; the YKL-40 level was elevated by 56%, while the average COMP concentration showed no change after the run. However, there was variation between the runners; COMP levels remained unaltered or slightly decreased in some of the runners, while they increased in the others, the maximal individual increase being 493.6 ng/ml. Accordingly, there are reports on increased and decreased levels of COMP following joint loading [5]–[7], [9], [10]. In general, our results highlight the marathon related increase in MMP-3 and YKL-40 which are recognized biomarkers of cartilage degradation associated with arthritis.

Adipokines leptin, adiponectin and resistin have recently been associated with inflammation and cartilage destruction in arthritis [25]. On the other hand, the length and energy expenditure in a single workout have been demonstrated to exert acute effects on adipokine levels [26]. Those series of knowledge led us to measure the changes in adipokine levels during a marathon race. To our knowledge, this is the first study to investigate the effects of marathon running on adiponectin and resistin levels. The race appeared to have significant effects on those adipokines: during the marathon, the resistin levels more than doubled and an increase was seen also in the adiponectin levels. Interestingly, the marathon induced elevation in the resistin concentrations was associated with the increases in MMP-3 and YKL-40 levels. Further, a high pre-marathon resistin level predicted a larger increase in YKL-40 during the run.

Resistin was initially named after its ability to evoke insulin resistance in rodents, but in humans the relation between resistin and insulin resistance or BMI is less clear [14]. A recent study revealed that the effects of this mainly macrophage derived adipocytokine are mediated after its binding to the Toll-like receptor 4 (TLR4) [27], which is the receptor known also to mediate many of the effects of bacterial products in the innate immune response. This finding indicates resistin as a proinflammatory cytokine. Previous studies have shown that resistin indeed has proinflammatory and cartilage destructing properties in joints: resistin was reported to induce synovial inflammation and cartilage destruction when injected into the knee joints of healthy mice [28], to enhance MMP-1, MMP-13 and ADAMTS expression in human primary chondrocytes [29], to upregulate PGE_2_, IL-6 and IL-8 production in mouse cartilage [30] and to decrease production of proteoglycan in mouse and in human cartilage [30]. To our knowledge, there are no previous studies reporting immediate effects of long-term exercise on resistin levels following long-term exercise, but the study of Perseghin et al. [31] reported elevated resistin concentrations in middle-distance and marathon runners, but not in the sprinters when compared to lean, young sedentary individuals. We report here that resistin levels doubled during running a marathon and that was associated with cartilage load/injury. Resistin is produced primarily by macrophages and within joints, resistin is also expressed in resident macrophage-like cells in synovium as well as in osteoblasts and osteoclasts [25], [32]. These cellular sources might contribute to the marathon induced increases locally within joints, which may then leak from the joint into the circulation. In fact, resistin levels in synovial fluid and in serum have been shown to increase in patients following knee injury [30] suggesting that marathon related mechanical joint load and microtrauma could contribute to the increased circulating resistin levels found in the present study. However, increased systemic resistin production cannot be excluded.

Adiponectin levels are low in obese patients, and patients with impaired glucose tolerance it has been reported to improve insulin sensitivity and exert antiatherogenic properties [14], [33], [34]. In addition, adiponectin has recently been reported to regulate inflammatory responses in relation to arthritis as well as cartilage metabolism. In OA, adiponectin has been shown to mediate cartilage destruction by enhancing the production of proinflammatory and cartilage destructing mediators NO, IL-6, MMP-1, MMP-3 and MMP-13 in primary chondrocytes and in cartilage. Further, adiponectin has also been found to associate with signs of cartilage destruction *in vivo*, i.e. with biomarkers COMP and MMP-3 and with radiographic severity of OA [15], [25]. In humans, adiponectin is primarily produced by adipocytes, but it is also expressed in skeletal muscle cells, cardiac myocytes and endothelial cells [14] and released from articular cartilage [15]. The present study reports a slight increase in adiponectin levels immediately after a marathon race and based on the present data it is not possible to determine the origin of the increased adipokine levels: significant metabolic changes take place both in the adipose tissue and in the muscle as well as in the cartilage. Interestingly, exercise has been shown to rapidly activate PPARγ in skeletal muscle, but recently also in monocytes (within 1.5–3 h of exercise) [35]. Further, Banga et al. showed that PPARγ activator pioglitatzone enhanced the secretion of adiponectin at 4 h timepoint, having no effect on adiponectin mRNA levels [36]. Result suggests that PPARγ activation has rapid posttranslational effects on e.g. adiponectin assembly and secretion. Exercise-induced PPARγ activation could thus contribute to the reported adiponectin increase. Since exercise is known to increase insulin sensitivity [37], it would be tempting to speculate that the elevated adiponectin level is involved in mediating this effect. Previous data on the effects of relatively long-term exercise on adiponectin levels is yet conflicting: apparently adiponectin levels did not exhibit changes following a 60-minute stationary cycle ergometry session in a healthy, moderately active group of 8 males and 8 females [38] or following a 120 min cycling session at 50% VO_2_max (maximal oxygen uptake) in group of 10 active males [39]. However, intensity and duration of the marathon run may explain the detected increase in adiponectin levels in the present study.

Leptin is a regulator of energy balance and leptin deficiency in experimental animals leads to hyperphagia and morbid obesity. However, increased blood levels of leptin in obese subjects fail to induce the expected responses, i.e. increased energy expenditure, and reduced food intake and lowering of body weight indicative of the appearance of leptin resistance [14]. Since leptin levels fall rapidly in response to fasting, the nutritional conditions present during intense exercise may account for the declines in leptin levels reported after long-term exercise [20]–[22]. Indeed, Koistinen et al. [19] showed that leptin levels remain unchanged if the marathon is run without fasting, but leptin levels do decrease after running a marathon under fasting conditions. Our study supports this finding as leptin levels remained unchanged immediately following this marathon where we imposed no restrictions on the calorie-intake during the race. According to recent findings, leptin appears also as an attractive candidate to link obesity, inflammatory mechanisms and osteoarthritis as it has been shown to enhance the production of proinflammatory cytokines and destructive mediators of OA, like NO, IL-6, prostaglandin E_2_ (PGE_2_) and MMPs by articular chondrocytes [25].

Since adipokines are regulatory factors in inflammation and in cartilage destruction [25] we investigated the possible associations between concentrations of resistin, adiponectin and leptin and the biomarkers MMP-3, YKL-40 and COMP following marathon run. The marathon induced changes in the resistin level were associated with increases in those of MMP-3 and YKL-40. Since it is known that the TLR4 is a receptor for resistin and that other activators of TLR4, like lipopolysaccharide (LPS), enhance production of NO, MMPs and other mediators of cartilage degradation [40], [41], one could speculate that resistin might play a role in the marathon induced inflammatory response and contribute to the subsequent catabolic effects on cartilage. We also found that pre-marathon adipokine levels were associated with the changes detected in biomarkers reflecting cartilage turnover and/or damage – a high pre-marathon resistin level was associated with a larger increase in the YKL-40 level, while low adiponectin and high leptin levels were related to a greater increase in the COMP concentration. These findings likely depict the interacting metabolic and inflammatory properties of the adipokines [14].

## Conclusions

The present results highlight the widespread impact of running a marathon on cartilage metabolism and imply that loading frequency increases cartilage turnover/damage: the faster the marathon was run, the greater the increase in the MMP-3 level. As an original finding, we report the effects of running a marathon on two adipokines, adiponectin and resistin which are both associated with cartilage destruction in inflammatory arthritis and OA: resistin levels were more than doubled after the run and an increase was seen also in the adiponectin levels. Interestingly, the marathon-induced elevation in the resistin concentration was associated with increases in biomarkers of cartilage degradation (MMP-3 and YKL-40) and further, a high pre-marathon resistin concentration predicted a larger increase in the YKL-40 level. Additional research is needed to clarify whether there is some causality between resistin and cartilage degradation and whether adipokines contribute to the marathon related increased risk of osteoarthritis [3].
